# Identification of Cysteine Ubiquitylation Sites on the Sec23A Protein of the COPII Complex Required for Vesicle Formation from the ER

**DOI:** 10.2174/1874091X01711010036

**Published:** 2017-04-28

**Authors:** Giuseppina Amodio, Luigi Margarucci, Ornella Moltedo, Agostino Casapullo, Paolo Remondelli

**Affiliations:** 1 Dipartimento di Medicina, Chirurgia e Odontoiatria “Scuola Medica Salernitana”, Università degli Studi di Salerno, 84084 Baronissi-Salerno, Italy; 2 Dipartimento di Farmacia, Università degli Studi di Salerno, 84034 Fisciano-Salerno, Italy

**Keywords:** Ubiquitylation, Sec23a, COPII, ERES, Vescicular transport

## Abstract

**Background:**

COPII is a multiprotein complex that surrounds carrier vesicles budding from the Endoplasmic Reticulum and allows the recruitment of secretory proteins. The Sec23a protein plays a crucial role in the regulation of the dynamics of COPII formation ensuring the proper function of the secretory pathway.

**Objective:**

Since few evidences suggest that ubiquitylation could have a role in the COPII regulation, the present study was aimed to establish whether the Sec23a component of the vesicular envelope COPII could be ubiquitylated

**Method:**

Sec23a ubiquitylation was revealed by co-immunoprecipitation experiments. Recombinant Sec23a was gel-purified and analyzed by mass spectrometry subjected to trypsin proteolysis. Signature peptides were identified by the presence of Gly–Gly remnants from the C-terminus of the ubiquitin attached to the amino acid residues of the substrate. Recombinant Sec23a proteins bearing mutations in the ubiquitylation sites were used to evaluate the effect of ubiquitylation in the formation of COPII

**Results:**

We identified two cysteine ubiquitylation sites showed at position 432 and 449 of the Sec23a protein sequence. Interestingly, we revealed that the amino acid residues of Sec23a joined to ubiquitin were cysteine instead of the conventional lysine residues. This unconventional ubiquitylation consists of the addition of one single ubiquitin moiety that is not required for Sec23a degradation. Immunofluorescence results showed that Sec23a ubiquitylation might influence COPII formation by modulating Sec23a interaction with the ER membrane. Presumably, this regulation could occur throughout continual ubiquitylation/de-ubiquityliation cycles.

**Conclusion:**

Our results suggest a novel regulatory mechanism for the Sec23a function that could be crucial in several pathophysiological events known to alter COPII recycling

## 1 INTRODUCTION

Secretory proteins exit the Endoplasmic Reticulum (ER) by entering onto vesicular carriers that form from specific membrane domains termed as ER Exit Sites (ERESs) [1-6]. At the ERESs, the Sec16 protein defines the location for the Coat Protein Complex II (COPII) assembly [7-12].

COPII is a multiprotein complex that surrounds budding vesicles and allows the recruitment of secretory proteins [13, 14]. The COPII envelope is made of five proteins: Sar1, Sec13-Sec23 dimer, Sec24 and Sec31dimer. COPII vesicles formation begins with the conversion of Sar1-GDP to Sar1-GTP via the GTP Exchange Factor (GEF) Sec12. Sar1-GTP inserts its N-terminal amphipathic helix into the cytoplasmic side of the ER membrane bilayer and initiates membrane curvature [15-18]. The Sec23–Sec24 dimer interacts with Sar1 and the ER membrane and forms an ‘inner coat’ that increases membrane curvature and, throughout Sec24 proteins, recruits cargo proteins into carrier vesicles [13, 18-25]. Vesicle formation is completed by the progressive polymerization of the Sec13–Sec31 dimer that forms the “outer coat” of COPII [26, 27].

Sec23a plays a crucial role in the regulation of the dynamics of COPII formation [28]. When the vesicle embedding is completed, Sec23a initiates vesicle un-coating by stimulating Sar1 to hydrolyse GTP ([15, 17, 29]. GDP-bound Sar1 pulls the amphipathic helix out of the vesicle membrane and this event triggers the disassembly of COPII and the recycling of the envelope subunits for the following round of vesicle formation [16, 30].

Ubiquitylation is a multistep enzymatic process that results in the attachment of ubiquitin, or chains of ubiquitin, to mark proteins for proteasomal degradation. Besides, ubiquitylation is involved in a number of cellular activities including cell division, differentiation, signal transduction and protein trafficking [31]. In particular, protein ubiquitylation can be used as a signal for the endocytosis mediated degradation of membrane proteins in order to regulate the functions of signalling receptors, ion channels, transporters [32-36].

Few evidences suggest that ubiquitylation could have a role in the COPII function. In budding yeast, Sec23 undergoes two rounds of ubiquitylation/de-ubiquitylation events that are important for the correct function of the yeast secretory pathway [37]. More interestingly, it has been shown that mono ubiquitylation of the COPII-component SEC31 controls the size of COPII coats and is critical for collagen export [38].

In the present report, we showed that the Sec23a component of COPII presents unconventional cysteine ubiquitylation sites. We suggest that such a modification could play a role in the formation of COPII at the ERESs.

## 2 MATERIALS AND METHODS

### 2.1 Cell Cultures and Drugs Treatments

Human hepatoma Huh7 cells and Human embryonic kidney cells 293T were maintained in high glucose DMEM (Thermo Fisher-Gibco) supplemented with 10% FBS (Thermo Fisher-Gibco), 2 mM L-glutamine (Lonza) and antibiotics (100 U/ml penicillin and 100 µg/ml streptomycin; Lonza). When indicated, actively growing cells were incubated either with 300 nM thapsigargin (TG, Sigma-Aldrich), 10 µM MG132 (Sigma-Aldrich), or 10 µg/ml Cycloheximide (CHX: Sigma-Aldrich). For transient transfection, FuGENE HD transfection reagent (Promega) was used according to the manufacturer’s instructions.

### 2.2 Construction of Plasmids

Human Sec23a cDNA was obtained by One-Step RT-PCR (Thermo Fisher-Invitrogen) performed on HeLa total RNA with gene specific primers spanning from nucleotide -26 to +2622 of Sec23a mRNA (5’-cgcaga aataagaatcaaactcc-3’ and 5’-tagagcaatatctgttggtttcc -3’). From this cDNA the full length coding sequence of Sec23a (from +1 to + 2306 bp) was amplified and subcloned in the HindIII-XbaI sites (primers: 5’-cccaagcttatgacaacctat ttggaattc-3’ and 5’-gctctagaattagcacttcaagcagc-3’) of the p3xFLAG-CMV-7.1 vector (Sigma-Aldrich) or in the KpnI-XbaI sites (primers: 5’-ggggtaccatgacaacctatttggaattg-3’ and 5’-gctctagaattagcacttcaagcagc-3’) of the pEGFP-C1 vector (Clontech) to obtain the p3xFLAG-Sec23a and the pEGFP-Sec23a expressing constructs respectively. The pHA-Ubiquitin vector was previously described [39]. Cysteine 432 and 449 were changed to alanine by site directed mutagenesis on the pEGFP-Sec23a construct according to the QuickChange kit (Agilent Technologies) instructions. Mutations were inserted by using the following primers: 5’-caattctaaaggacccgctgtgtctgaaaatgag-3’ plus 3’-cagtgagttaagatttcctggg-5’ for C432A mutation and 5’-catgtcagtggaagatagctggacttagtcccactac-3’ plus 5’-cttccactgacatgtgccacc-3’ for C449A mutation. All the sequences of the recombinant plasmids described were verified by sequencing.

### 2.3 Co-Immunoprecipitation

10 cm dish-cultured HuH7 or Hek 293 T cells were co-transfected with either the p3xFLAG-Sec23a or the p3XFLAG empty vector plus the same DNA amount of the pcDNA3/HA-Ubiquitin expression vector [40]. At 48 hours, the cells were subjected to drug treatments, as indicated, before to be harvested in the lysis buffer (10 mM Tris-HCl pH 7.4, 150 mM NaCl, 1 mM EDTA pH 8.0, 1% Triton X-100) supplemented by protease inhibitor cocktail (Roche). Equal amounts of cell lysates were incubated with mouse anti-HA monoclonal antibody (F-7; Santa Cruz Biotechnology) overnight at 4°C, followed by incubation with protein A-Sepharose beads (GE Healthcare) for 1 hour at 4°C. The immune complexes were analysed by SDS-PAGE (10%) and revealed by mouse anti-FLAG monoclonal antibody (M2, Sigma-Aldrich), mouse anti-HA antibody and mouse monoclonal anti-ʏ tubulin (Santa Cruz Biotechnology). HRP-conjugated goat anti-mouse antibody (Sigma-Aldrich) was used as the secondary antibody.

### 2.4 Mass Spectrometric Analysis of Sec23a Ubiquitylation Sites

Actively growing Huh7 cells were transfected with 8 µg of 3X-FLAG-Sec23a and 48 h after transfection were lysed in lysis buffer (10 mM Tris-HCl pH 7.4, 150 mM NaCl, 1 mM EDTA pH 8.0, 1% Triton X-100) complemented with protease inhibitor cocktail (Roche). Lysates were immunoprecipitated by the anti-FLAG agarose beads (Sigma-Aldrich), subjected to SDS-PAGE and revealed by comassie staining. The gel band strongly enriched by FLAG conjugated beads was cut out and subjected to *in situ* protein digestion as described in Shevschenko protocol [41]. Briefly, gel slices were reduced and alkylated using 1,4-dithiothreitol (10 mM) and iodoacetamide (54 mM) respectively, then washed and rehydrated in trypsin solution (10 ng/μl) in ice for 1h. After the addition of 30 μl ammonium bicarbonate (10 mM, pH 7.5), samples were digested overnight at 37°C. The supernatants were collected and peptides were extracted by the gel slices using 100% CH_3_CN. Finally, the supernatant was collected and both were combined. All the peptides were dried out and dissolved in 10% FA before mass spectrometry analysis. 5 μl of the obtained peptide mixture was injected into a nano Acquity LC system (Waters Corp. Manchester, United Kingdom). The peptides were separated using a 1.7 µm BEH C-18 column (Waters Corp. Manchester, United Kingdom) at a flow rate of 400 nl/min. Peptide elution was achieved with a linear gradient from 15 to 50% (solution A: 95% H_2_O, 5% CH_3_CN, 0.1% FA; solution B: 95% ACN, 5% H_2_O, 0.1% FA) in 55 min. MS and MS/MS data were acquired using a Q-TOF Premier mass spectrometer (Waters Corp., Micromass, Manchester, United Kingdom). Five most intense doubly and triply charged peptide-ions were automatically chosen by the MassLynx software and fragmented. After mass spectrometric measurements, data were automatically processed by ProteinLynx software to generate peak lists for protein identifications. Database searches were carried out with MASCOT server. The SwissProt database (release 2010_11 of 02 Nov 10, 522019 sequences, 184241293 residues) was searched, allowing 2 missed cleavages, carbamidomethyl (C) as fixed modification o and oxidation (M) and phosphorylation (ST) as variable modifications. The peptide tolerance was set to 80 ppm and the MS/MS tolerance to 0.8 Da.

### 2.5 Immunofluorescence Analysis

Huh7 cells seeded on glass cover slips were transfected with wild type or mutant pEGFP-Sec23a. 48 hours after transfection, coverslips were washed in cold PBS, fixed for 10 min in PBS-4% paraformaldehyde and incubated for 30 min in PBS containing 0.5% BSA, 0.005% saponin and 50 mM NH4Cl at room temperature. The endogenous Sec16a was labelled with rabbit polyclonal Sec16a primary antibody (KIAA0310, Bethyl Laboratories) and goat anti rabbit Cy3-coniugated secondary antibody (GE Healthcare). Coverslips were mounted with the Prolong AntiFade kit (Molecular Probes, Invitrogen). Images were collected using a laser scanning confocal microscope (Leica TCS SP5 II) equipped with a plan Apo 63X, NA 1.4 oil immersion objective lens. The COPII coats labelled with exogenous Sec23a-GFP were measured in every sample by counting the GFP fluorescent spots in two in-focus z-planes by using Image J software on a minimum of 50 cells. The percent of Sec23a-GFP pixels colabeling with Sec16a pixels was quantified by Leica colocalization analysis tool in two in-focus z-planes on a minimum of 50 different cells.

## 3 RESULTS

### 3.1 Sec23a is Constitutively Ubiquitylated

**Fig. (1):**
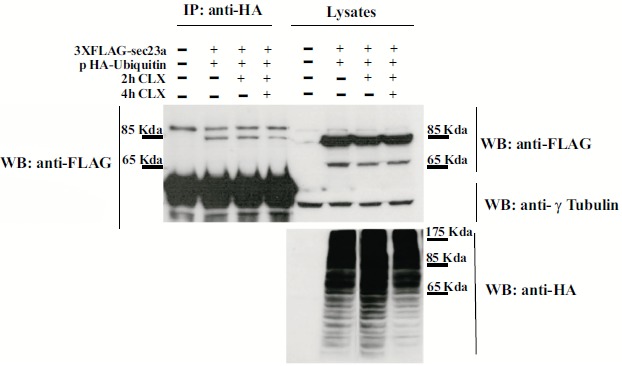
**Ubiquitylation of Sec23a**. Huh7 cells were co-transfected with the pHA-Ubiquitin expression vector and the 3xFLAG-Sec23a vector for 48 h and then subjected to cycloheximide treatment for the indicated time. Cell lysates were immunoprecipitated by a mouse monoclonal anti-HA antibody to selectively pull down ubiquitinated proteins and then the 3xFLAG-Sec23a was revealed by a mouse monoclonal anti-FLAG antibody. Total lysates were checked by Western Blot with the indicated antibodies as control of the input samples.

To test whether Sec23a could be ubiquitylated, we performed transfection experiments by using a plasmid vector expressing the ubiquitin protein fused to the hemo agglutinin (HA) epitope (pHA-Ubiquitin) and a plasmid vector encoding the recombinant Sec23a protein tagged with the 3xFLAG epitope (Sec23a-3xFLAG). In the immunoprecipitation assays, the anti HA antibody was used to pull-down ubiquitylated proteins and the anti-FLAG antibody to reveal the Sec23a-3xFLAG recombinant protein by western blotting (WB). Cell lysates of co-transfected cells analyzed by WB showed the presence of either the recombinant Sec23a or of a pattern of ubiquitin-coniugated proteins, as revealed by the anti-FLAG and anti-HA antibodies, respectively (Fig. **1**). Instead, WB analysis performed on the pulled-down lysates and revealed by the anti-HA antibody showed the presence of the Sec23a-3xFLAG protein Fig. (**1**), indicating that Sec23a is conjugated to ubiquitin. In addition, WB analyses showed a single form of the Sec23a-3xFLAG having a slightly higher molecular weight, which suggests that Sec23a might be conjugated to a single ubiquitin moiety. Remarkably, in the cells treated with the protein synthesis inhibitor cycloexymide, the amount of Sec23a-3xFLAG recombinant protein bound to ubiquitin remained unchanged at 2 h of cycloexymide exposure and showed only a minor decrease at 4 h treatment, implying that ubiquitylation of Sec23a is not required for proteasomal degradation.

### 3.2 Sec23a is Ubiquitylated at Cysteine 432 And Cysteine 449

**Fig. (2):**
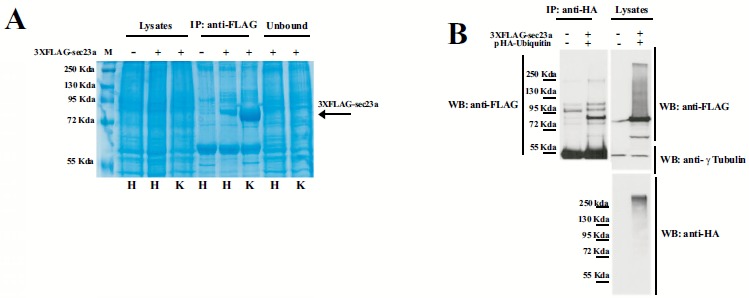
**Ubiquitylation of Sec23a in Hek293T cells.** (A) SDS-PAGE and Coomassie-staining of Huh7 (H) and Hek293T (K) cells transiently transfected with 3X-FLAG-Sec23a at 48 h of expression. Total extracts (Lysates), the FLAG-immunoprecipitated proteins and the unbound proteins are showed as steps of the 3xFLAG-Sec23a purification. (B) Hek293T cells were co-transfected with the pHA-Ubiquitin expression vector and the 3xFLAG-Sec23a vector. Cell lysates were immunoprecipitated and checked by Western Blot with the indicated antibodies as in (Fig. **1**).

**Fig. (4):**
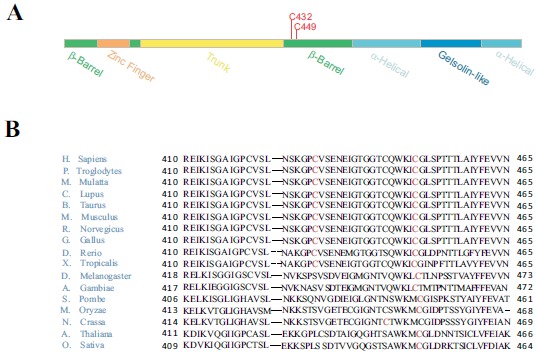
**Domain structure and sequence homology of Sec23a proteins**. (A) Schematic representation of the structural domains of Sec23a (B) Protein multiple alignment obtained by HomoloGene (https://www.ncbi.nlm.nih.gov/homologene**)** of Sec23a proteins derived from various species. Conserved Cys 432 and Cys 449 are highlighted in red.

To further investigate Sec23a ubiquitylation, we used a mass spectrometric approach. This method has been used to identify ubiquitylation sites, based on the fact that trypsin proteolysis of an ubiquitylated protein produces a signature peptide. This signature peptide is characterized by a Gly–Gly remnant from the C-terminus of the ubiquitin attached to the lysine residues of the substrate and can be identified by a mass shift in the modified residue or peptide [40, 42]. Thus, we over-expressed 3X-FLAG-Sec23a in Hek293 cells by transient transfection. Following that, the exogenous protein was purified from the total protein extract by affinity chromatography with anti-FLAG agarose resin and subjected to SDS-PAGE (Fig. **2A**). The proteins were revealed by coomassie brilliant blue staining Fig. (**2B**) and the 3X-FLAG-Sec23a specific band was extracted from the gel, subjected to in gel-trypsin digestion and the resulting peptides analyzed by mass spectrometry. As shown in Fig. (**3**), we found two peptides modified by Gly-Gly remnants (D) bound to cysteine residues. Mass fragmentation of these peptides disclosed the punctual site of ubiquitylation at cysteines 432 and 449, that are localized in the second β-Barrel domain of Sec23a protein and highly conserved among higher Eukaryiota (Fig. **4**). Interestingly, the β-Barrel domain is involved in the binding of Sec23a to the ER membrane [23, 43] suggesting that the ubiquitylation of this domain on cysteine 432 and 449 could affect the ability of Sec23a to bind to the ERESs during COPII coat formation.

### 3.3 Sec23a Ubiquitylation Influences Formation of COPII at the Eress

**Fig. (5):**
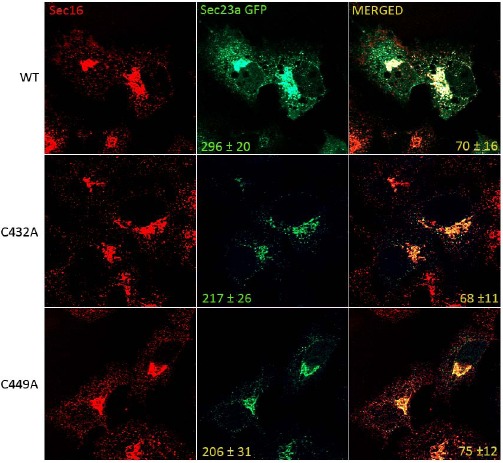
**Sec23a ubiquitylation affects COPII vesicles formation.** Huh7 cells seeded on glass coverslips were transiently transfected with the wilde type Sec23a-GFP or the mutated isoforms (C432A-Sec23a-GFP or C449A-Sec23a-GFP) and after 48 h of transfection processed for immunofluorescence with Sec16 specific antibody. The mean number of Sec23a-GFP positive spots (mean ± SD) and the percent of Sec23a-GFP pixels colabeling with Sec16a pixels (mean ± SD) are reported in the Sec23a-GFP panels and in the merged panels respectively.

To test whether cysteine ubiquitylation of Sec23a could have a role in the control of COPII assembly at the ERESs, we compared by immunofluorescence the level of the recombinant Sec23a-GFP protein localization with respect to that of the mutant Sec23a-GFP forms carrying either C432A mutation (C432A-Sec23a-GFP) or the C449A mutation (C449A-Sec23a-GFP). In the Huh7 transfected cell, endogenous Sec16 protein was used as reference protein for the ERESs. Our results showed that Sec16 is localized in punctuate structures disseminated all through the cytoplasm (Fig. **5**). These spots represent the amount of potential ERESs located at the ER membranes of Huh7 cells. In agreement with previous results [44], the dots labelled by the wild type Sec23a-GFP protein colocalized only to some degree with the ones of Sec16 (70±16%). Interestingly, quantitative analysis showed that the average number of dots corresponding to COPII budding vesicles and containing wild type Sec23a-GFP was 296±20. In contrast, the number of COPII labelled by the mutant recombinant proteins declined to 217±26 for the C432A-Sec23a-GFP mutant protein and 206±31 for the C449A-Sec23a-GFP protein. Curiously, the C432A-Sec23a-GFP co-labelled with Sec16 quite to the same extent (68±11) of the wild type Sec23a-GFP protein, while the C449A-Sec23a-GFP co-localized with Sec16 to little higher rate (75±12%) compared to the un-mutated recombinant protein (70±16%).

## 4 DISCUSSION

The major result of our study is the identification of two cysteine ubiquitylation sites revealed at position 432 and 449 of the Sec23a protein sequence. More interestingly, our results suggest that this unconventional ubiquitylation is not required for Sec23a protein degradation. This hypothesis is in agreement with the several evidences showing that protein mono-ubiquitylation doesn’t necessarily support proteasome degradation and can be involved in other functions including vesicular transport.

Even more remarkable is the finding that the amino acid residues of Sec23a modified by the ubiquitylation are cysteine instead of the conventional lysine residues. Cysteine ubiquitylation can occur throughout a thioesterification. Few evidences are reported demonstrating that such a modification is important for the cell physiology. For instance, it has been described, in yeast as well as in mammals, that the peroxisome import receptor Pex5p is mono-ubiquitylated on a conserved cysteine [45]. Furthermore, cysteine ubiquitylation of Pex5p is required for release of the receptor from the peroxisome membrane to allow the receptor recycling for further rounds of import.

In our case we found that ubiquitylated cysteines 432 and 449 are highly conserved and both localized in the β-barrel domain of Sec23a that is known as the membrane binding domain of Sec23a [23, 43]. Thus, it is conceivable that cysteine ubiquitylation of Sec23a, as suggested by immunofluorescence results, might influence COPII formation at the ERESs by modulating Sec23a interaction with the ER membrane. Presumably, this regulation could occur throughout continual ubiquitylation/de-ubiquitylation cycles.

Many signaling pathways can control COPII function by modulating its formation and/or recycling by modifying components of the ER export machinery [46-49].

Cystein ubiquitylation of Sec23a could be involved in several pathophysiological events well-known to alter COPII recycling characterized by overload of cargo proteins [50, 51]. Intriguingly, in mammalian cells, acute cargo load reduces the number of ERESs, while continual protein load increases the amount of ERESs [52]. Moreover, in our previous studies we showed that the ER calcium transport inhibitor thapsigargin, which perturbs calcium homeostasis, modulates the expression of ER resident factors and induces ER stress [53]. Under ER stress, TG can decrease COPII assembly by modifying Sec23a cycling at the ERESs [54] and, as a consequence, the anterograde transport of cargo proteins [55-58]. Interestingly, preliminary results obtained in our lab revealed that level of Sec23a ubiquitylation decreased in TG induced cells suggesting that ER stress could regulate the rate of Sec23a ubiquitylation/de-ubiquitylation cycles.

Finally, level of Sec23a ubiquitylation should be evaluated in association with other factors that can influence COPII assembly such as the p125 protein at the ERESs [59], the Ca(2+)-dependent protein ALG2 [60, 61], the TFG1 oncoprotein [62] or in the genetic disorder Cranio-Lenticulo-Sutural Dysplasia (CLSD) caused by a SEC23A mutation that leads to abnormal endoplasmic-reticulum-to-Golgi trafficking [63].

## Figures and Tables

**Fig. (3):**
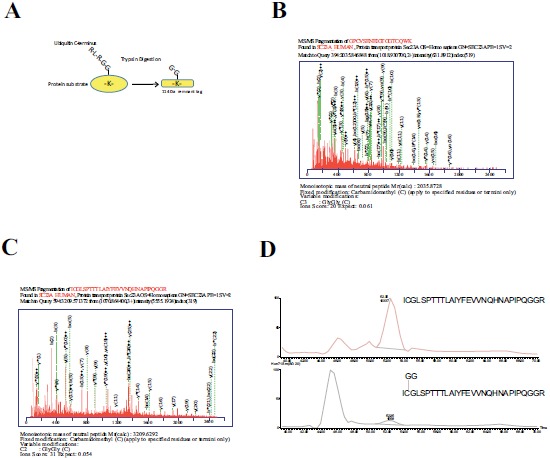
**Sec23a is ubiquitylated on cys432 and cys449.** (A) Schematic representation of the Gly-Gly remnant left on ubiquitinated residues after trypsin digestion of the C-terminus of the linked ubiquitin (B;C) Output view of the software used for the search of the Gly-Gly modified peptides of Sec23a. The sequence and the MS/MS fragmentation peaks of the peptides containing the Gly-Gly-Cys 432 (B) and Gly-Gly-Cys 449 (C) are shown. (D) Extracted ion chromatograms corresponding to the Cys-modified peptide species.

## References

[1] Palade G. (1975). Intracellular aspects of the process of protein synthesis.. Science.

[2] Orci L., Ravazzola M., Meda P., Holcomb C., Moore H.P., Hicke L., Schekman R. (1991). Mammalian Sec23p homologue is restricted to the endoplasmic reticulum transitional cytoplasm.. Proc. Natl. Acad. Sci. USA.

[3] Aridor M., Balch W.E. (1996). Principles of selective transport: coat complexes hold the key.. Trends Cell Biol..

[4] Bannykh S.I., Rowe T., Balch W.E. (1996). The organization of endoplasmic reticulum export complexes.. J. Cell Biol..

[5] Tang B.L., Wang Y., Ong Y.S., Hong W. (2005). COPII and exit from the endoplasmic reticulum.. Biochim. Biophys. Acta.

[6] Hughes H., Stephens D.J. (2008). Assembly, organization, and function of the COPII coat.. Histochem. Cell Biol..

[7] Supek F., Madden D.T., Hamamoto S., Orci L., Schekman R. (2002). Sec16p potentiates the action of COPII proteins to bud transport vesicles.. J. Cell Biol..

[8] Mironov A.A., Mironov A.A., Beznoussenko G.V., Trucco A., Lupetti P., Smith J.D., Geerts W.J., Koster A.J., Burger K.N., Martone M.E., Deerinck T.J., Ellisman M.H., Luini A. (2003). ER-to-Golgi carriers arise through direct en bloc protrusion and multistage maturation of specialized ER exit domains.. Dev. Cell.

[9] Zeuschner D., Geerts W.J., van Donselaar E., Humbel B.M., Slot J.W., Koster A.J., Klumperman J. (2006). Immuno-electron tomography of ER exit sites reveals the existence of free COPII-coated transport carriers.. Nat. Cell Biol..

[10] Watson P., Townley A.K., Koka P., Palmer K.J., Stephens D.J. (2006). Sec16 defines endoplasmic reticulum exit sites and is required for secretory cargo export in mammalian cells.. Traffic.

[11] Bhattacharyya D., Glick B.S. (2007). Two mammalian Sec16 homologues have nonredundant functions in endoplasmic reticulum (ER) export and transitional ER organization.. Mol. Biol. Cell.

[12] Whittle J.R., Schwartz T.U. (2010). Structure of the Sec13-Sec16 edge element, a template for assembly of the COPII vesicle coat.. J. Cell Biol..

[13] Barlowe C., Orci L., Yeung T., Hosobuchi M., Hamamoto S., Salama N., Rexach M.F., Ravazzola M., Amherdt M., Schekman R. (1994). COPII: a membrane coat formed by Sec proteins that drive vesicle budding from the endoplasmic reticulum.. Cell.

[14] Shaywitz D.A., Espenshade P.J., Gimeno R.E., Kaiser C.A. (1997). COPII subunit interactions in the assembly of the vesicle coat.. J. Biol. Chem..

[15] Yoshihisa T., Barlowe C., Schekman R. (1993). Requirement for a GTPase-activating protein in vesicle budding from the endoplasmic reticulum.. Science.

[16] Barlowe C., Schekman R. (1993). SEC12 encodes a guanine-nucleotide-exchange factor essential for transport vesicle budding from the ER.. Nature.

[17] Bielli A., Haney C.J., Gabreski G., Watkins S.C., Bannykh S.I., Aridor M. (2005). Regulation of Sar1 NH2 terminus by GTP binding and hydrolysis promotes membrane deformation to control COPII vesicle fission.. J. Cell Biol..

[18] Lee M.C., Orci L., Hamamoto S., Futai E., Ravazzola M., Schekman R. (2005). Sar1p N-terminal helix initiates membrane curvature and completes the fission of a COPII vesicle.. Cell.

[19] Miller E.A., Beilharz T.H., Malkus P.N., Lee M.C., Hamamoto S., Orci L., Schekman R. (2003). Multiple cargo binding sites on the COPII subunit Sec24p ensure capture of diverse membrane proteins into transport vesicles.. Cell.

[20] Miller E., Antonny B., Hamamoto S., Schekman R. (2002). Cargo selection into COPII vesicles is driven by the Sec24p subunit.. EMBO J..

[21] Hicke L., Yoshihisa T., Schekman R. (1992). Sec23p and a novel 105-kDa protein function as a multimeric complex to promote vesicle budding and protein transport from the endoplasmic reticulum.. Mol. Biol. Cell.

[22] Matsuoka K., Orci L., Amherdt M., Bednarek S.Y., Hamamoto S., Schekman R., Yeung T. (1998). COPII-coated vesicle formation reconstituted with purified coat proteins and chemically defined liposomes.. Cell.

[23] Bi X., Corpina R.A., Goldberg J. (2002). Structure of the Sec23/24-Sar1 pre-budding complex of the COPII vesicle coat.. Nature.

[24] Bi X., Mancias J.D., Goldberg J. (2007). Insights into COPII coat nucleation from the structure of Sec23.Sar1 complexed with the active fragment of Sec31.. Dev. Cell.

[25] Wendeler M.W., Paccaud J.P., Hauri H.P. (2007). Role of Sec24 isoforms in selective export of membrane proteins from the endoplasmic reticulum.. EMBO Rep..

[26] Fath S., Mancias J.D., Bi X., Goldberg J. (2007). Structure and organization of coat proteins in the COPII cage.. Cell.

[27] Stagg S.M., Gürkan C., Fowler D.M., LaPointe P., Foss T.R., Potter C.S., Carragher B., Balch W.E. (2006). Structure of the Sec13/31 COPII coat cage.. Nature.

[28] Lord C., Bhandari D., Menon S., Ghassemian M., Nycz D., Hay J., Ghosh P., Ferro-Novick S. (2011). Sequential interactions with Sec23 control the direction of vesicle traffic.. Nature.

[29] Sato K., Nakano A. (2005). Dissection of COPII subunit-cargo assembly and disassembly kinetics during Sar1p-GTP hydrolysis.. Nat. Struct. Mol. Biol..

[30] Antonny B., Madden D., Hamamoto S., Orci L., Schekman R. (2001). Dynamics of the COPII coat with GTP and stable analogues.. Nat. Cell Biol..

[31] Mukhopadhyay D., Riezman H. (2007). Proteasome-independent functions of ubiquitin in endocytosis and signaling.. Science.

[32] Haglund K., Dikic I. (2012). The role of ubiquitylation in receptor endocytosis and endosomal sorting.. J. Cell Sci..

[33] Acconcia F., Sigismund S., Polo S. (2009). Ubiquitin in trafficking: the network at work.. Exp. Cell Res..

[34] Marchese A., Trejo J. (2013). Ubiquitin-dependent regulation of G protein-coupled receptor trafficking and signaling.. Cell. Signal..

[35] Eaton D.C., Malik B., Bao H.F., Yu L., Jain L. (2010). Regulation of epithelial sodium channel trafficking by ubiquitination.. Proc. Am. Thorac. Soc..

[36] Hislop J.N., von Zastrow M. (2011). Role of ubiquitination in endocytic trafficking of G-protein-coupled receptors.. Traffic.

[37] Cohen M., Stutz F., Belgareh N., Haguenauer-Tsapis R., Dargemont C. (2003). Ubp3 requires a cofactor, Bre5, to specifically de-ubiquitinate the COPII protein, Sec23.. Nat. Cell Biol..

[38] Jin L., Pahuja K.B., Wickliffe K.E., Gorur A., Baumgärtel C., Schekman R., Rape M. (2012). Ubiquitin-dependent regulation of COPII coat size and function.. Nature.

[39] Mauro C., Pacifico F., Lavorgna A., Mellone S., Iannetti A., Acquaviva R., Formisano S., Vito P., Leonardi A. (2006). ABIN-1 binds to NEMO/IKKgamma and co-operates with A20 in inhibiting NF-kappaB.. J. Biol. Chem..

[40] Kirkpatrick D.S., Weldon S.F., Tsaprailis G., Liebler D.C., Gandolfi A.J. (2005). Proteomic identification of ubiquitinated proteins from human cells expressing His-tagged ubiquitin.. Proteomics.

[41] Shevchenko A., Tomas H., Havlis J., Olsen J.V., Mann M. (2006). In-gel digestion for mass spectrometric characterization of proteins and proteomes.. Nat. Protoc..

[42] Peng J., Schwartz D., Elias J.E., Thoreen C.C., Cheng D., Marsischky G., Roelofs J., Finley D., Gygi S.P. (2003). A proteomics approach to understanding protein ubiquitination.. Nat. Biotechnol..

[43] Lederkremer G.Z., Cheng Y., Petre B.M., Vogan E., Springer S., Schekman R., Walz T., Kirchhausen T. (2001). Structure of the Sec23p/24p and Sec13p/31p complexes of COPII.. Proc. Natl. Acad. Sci. USA.

[44] Hughes H., Budnik A., Schmidt K., Palmer K.J., Mantell J., Noakes C., Johnson A., Carter D.A., Verkade P., Watson P., Stephens D.J. (2009). Organisation of human ER-exit sites: requirements for the localisation of Sec16 to transitional ER.. J. Cell Sci..

[45] Okumoto K., Misono S., Miyata N., Matsumoto Y., Mukai S., Fujiki Y. (2011). Cysteine ubiquitination of PTS1 receptor Pex5p regulates Pex5p recycling.. Traffic.

[46] Zacharogianni M., Kondylis V., Tang Y., Farhan H., Xanthakis D., Fuchs F., Boutros M., Rabouille C. (2011). ERK7 is a negative regulator of protein secretion in response to amino-acid starvation by modulating Sec16 membrane association.. EMBO J..

[47] Palmer K.J., Konkel J.E., Stephens D.J. (2005). PCTAIRE protein kinases interact directly with the COPII complex and modulate secretory cargo transport.. J. Cell Sci..

[48] Wang L., Lucocq J.M. (2007). p38 MAPK regulates COPII recruitment.. Biochem. Biophys. Res. Commun..

[49] Farhan H., Wendeler M.W., Mitrovic S., Fava E., Silberberg Y., Sharan R., Zerial M., Hauri H.P. (2010). MAPK signaling to the early secretory pathway revealed by kinase/phosphatase functional screening.. J. Cell Biol..

[50] Forster R., Weiss M., Zimmermann T., Reynaud E.G., Verissimo F., Stephens D.J., Pepperkok R. (2006). Secretory cargo regulates the turnover of COPII subunits at single ER exit sites.. Curr. Biol..

[51] Tabata K.V., Sato K., Ide T., Nishizaka T., Nakano A., Noji H. (2009). Visualization of cargo concentration by COPII minimal machinery in a planar lipid membrane.. EMBO J..

[52] Farhan H., Weiss M., Tani K., Kaufman R.J., Hauri H.P. (2008). Adaptation of endoplasmic reticulum exit sites to acute and chronic increases in cargo load.. EMBO J..

[53] Amodio G., Moltedo O., Monteleone F., DAmbrosio C., Scaloni A., Remondelli P., Zambrano N. (2011). Proteomic signatures in thapsigargin-treated hepatoma cells.. Chem. Res. Toxicol..

[54] Amodio G., Venditti R., De Matteis M.A., Moltedo O., Pignataro P., Remondelli P. (2013). Endoplasmic reticulum stress reduces COPII vesicle formation and modifies Sec23a cycling at ERESs.. FEBS Lett..

[55] Spatuzza C., Renna M., Faraonio R., Cardinali G., Martire G., Bonatti S., Remondelli P. (2004). Heat shock induces preferential translation of ERGIC-53 and affects its recycling pathway.. J. Biol. Chem..

[56] Renna M., Faraonio R., Bonatti S., De Stefano D., Carnuccio R., Tajana G., Remondelli P. (2006). Nitric oxide-induced endoplasmic reticulum stress activates the expression of cargo receptor proteins and alters the glycoprotein transport to the Golgi complex.. Int. J. Biochem. Cell Biol..

[57] Renna M., Caporaso M.G., Bonatti S., Kaufman R.J., Remondelli P. (2007). Regulation of ERGIC-53 gene transcription in response to endoplasmic reticulum stress.. J. Biol. Chem..

[58] Amodio G., Renna M., Paladino S., Venturi C., Tacchetti C., Moltedo O., Franceschelli S., Mallardo M., Bonatti S., Remondelli P. (2009). Endoplasmic reticulum stress reduces the export from the ER and alters the architecture of post-ER compartments.. Int. J. Biochem. Cell Biol..

[59] Shimoi W., Ezawa I., Nakamoto K., Uesaki S., Gabreski G., Aridor M., Yamamoto A., Nagahama M., Tagaya M., Tani K. (2005). p125 is localized in endoplasmic reticulum exit sites and involved in their organization.. J. Biol. Chem..

[60] Shibata H., Suzuki H., Yoshida H., Maki M. (2007). ALG-2 directly binds Sec31A and localizes at endoplasmic reticulum exit sites in a Ca2+-dependent manner.. Biochem. Biophys. Res. Commun..

[61] la Cour J.M., Schindler A.J., Berchtold M.W., Schekman R. (2013). ALG-2 attenuates COPII budding in vitro and stabilizes the Sec23/Sec31A complex.. PLoS One.

[62] Witte K., Schuh A.L., Hegermann J., Sarkeshik A., Mayers J.R., Schwarze K., Yates J.R., Eimer S., Audhya A. (2011). TFG-1 function in protein secretion and oncogenesis.. Nat. Cell Biol..

[63] Boyadjiev S.A., Fromme J.C., Ben J., Chong S.S., Nauta C., Hur D.J., Zhang G., Hamamoto S., Schekman R., Ravazzola M., Orci L., Eyaid W. (2006). Cranio-lenticulo-sutural dysplasia is caused by a SEC23A mutation leading to abnormal endoplasmic-reticulum-to-Golgi trafficking.. Nat. Genet..

